# Heparin Anticoagulation Responsiveness in a Coronary Care Unit: A Prospective Observational Study

**DOI:** 10.1111/j.1755-5922.2009.00076.x

**Published:** 2009

**Authors:** Faisal Alsayegh, Mona Al-Rasheed, Ali Al-Muhaini, Ekhlas Al-Humoud, Mona Al-Ostaz, Shaker A Mousa

**Affiliations:** 1 Faculty of medicine, Kuwait universityKuwait; 2 Mubarak Alkabeer hospital, Ministry of healthKuwait; 3 Farwaniya hospital, Ministry of healthKuwait; 4 The Pharmaceutical research Institute, Albany College of PharmacyAlbany, NY, USA

**Keywords:** Heparin resistance, Anticoagulant, Coronary care, Unstable angina, Myocardial infarction, Monitoring

## Abstract

The aim of this study is to assess the practice of heparin administration in real-life situations. This study was conducted at the coronary care unit (CCU) in one of the busiest hospitals in Kuwait; with special attention to the rate of heparin resistance, potential factor that may predict resistance or responsiveness and heparin related complications. A prospective observational study was conducted in Farwania hospital over a 4-month period; this study included 146 patients admitted to the CCU. Patients were treated with UFH according to a standard normogram. Several variables were collected and analyzed, including demographic data, initial diagnosis, activated partial thromboplastin time (APTT) on admission and at 6, 24, and 48 h after UFH administration, and any complications that occurred. A significant number of patients had subtherapeutic APTT at 6, 24, and 48 h (41.1%, 42.3%, and 46.7%, respectively). There were four factors that predicted heparin resistance, including race, gender, admitting diagnosis (unstable angina vs. acute myocardial infarction), and an APTT ratio of less than one on admission. There was no significant difference in the rate of development of complications among different groups. Heparin resistance is a common phenomenon especially in the first period of heparin therapy. Special attention should be given to some groups like females, patients admitted with unstable angina, and those with APTT below the normal range. Evidence based protocols for Heparin administration and monitoring must be adopted to prevent the risk of under or over anticoagulation.

## Introduction

Unfractionated heparin (UFH) is an antithrombotic agent used as a first line therapy in the management and prophylaxis of thromboembolic disorders and acute coronary syndromes [1]. Heparin is an indirect thrombin inhibitor, which complexes with antithrombin (AT, formerly known as AT III) and converts this circulating cofactor from a slow-to-rapid inactivator of thrombin, factor Xa, and to a lesser extent, factors XIIa, XIa, and IXa [2,3].

Heparin has a number of major limitations, including a narrow therapeutic window of adequate anticoagulation without running an increased risk of bleeding and a highly variable dose–response relation that requires monitoring by laboratory testing. Heparin resistance is a known phenomenon, especially in the early phase of heparin therapy, which is attributed to nonspecific binding of UFH to the endothelial cells and plasma proteins, active removal of UFH by the reticuloendothelial cells, and neutralization by platelet factor-4 [4,5]. This initial phase of heparin resistance may put patients at risk of progression of the underlying thrombotic process [6]. Therefore, close monitoring of the activated partial thromboplastin time (APTT) and heparin dose adjustment is recommended to reduce this risk. In Kuwait, as well as in the Middle East, UFH is the most widely used anticoagulant in the management of thromboembolic disorders and acute coronary syndromes. However, the practice of anticoagulation, potential practice pitfalls, and the possible factors that could influence the outcome have never been previously explored in studies from the Middle East.

## Methods

The study was conducted in Farwania hospital, a large community-based hospital in Kuwait serving a population of 700,000. The data were collected prospectively over a period of 4 months. The treating physician were blinded to data collection. The Institutional Review Board at Farwaniya Hospital approved the study. Informed consent was obtained from each participant.

### Inclusion Criteria

All patients admitted to the coronary care unit (CCU) with a diagnosis of myocardial infarction or unstable angina and requiring UFH were included in the study. Patients who were found later to have noncardiac chest pain but received UFH in the initial phase of admission to the CCU were also included.

### Exclusion Criteria

Patients who did not receive UFH during their stay in the CCU were excluded from the study.

### Primary Outcome

The primary outcome was to assess the number of patients who achieved therapeutic APTT with UFH therapy within the first 6, 24. and 48 h of their admission.

### Secondary Outcome

Assessment of any factors that may influence the achievement of a therapeutic range determined by APTT in the initial phase of the treatment, and examination of any potential complications that may result from under- or overanticoagulation with UFH such as recurrence of ischemia, progression of thrombus, pulmonary embolism (PE), stroke, death, and bleeding.

### Treatment

The dose of heparin was adjusted by treating physician according to a heparin normogram in which 5000 unit heparin bolus is given initially, followed by an intravenous infusion of heparin at a rate of 1000 units/h. Further readjustment of the dose depends on the APTT test result. APTT was measured every 6 h, and the dose of heparin was adjusted to keep APTT in the therapeutic range.

### Laboratory Monitoring

The anticoagulant effect of UFH is assessed by APTT. An APTT ratio of 1.5–2.5 times the mean was considered therapeutic in the local hospital laboratory. A subtherapeutic APTT was defined as an APTT ratio that is less than 1.5. Any ratio higher than 2.5 is considered overanticoagulated state.

APTT was recorded on admission and then at 6, 24, 48, and 72 h of starting UFH. Those patients who required a longer period of anticoagulation had their APTT recorded in the subsequent days, and the daily total dose of UFH is recorded.

### Statistical Analysis

Analysis of data is mainly descriptive using means and standard deviations or median and ranges. The Pearson Chi-square test was used to assess secondary outcomes.

## Results

At the end of the study period, 146 patients were included. Patient's characteristics and diagnosis on admission are summarized in table 1.

**Table 1:** Baseline characteristics

	Number (%)	Total
Sex		
Male	99 (67.8)	
Female	47 (32.2)	146(100%)
Age:		
Range	23–92 years	
Mean	52.51 years	
Racial background		
Kuwaiti Arabs	64 (43.8)	
Non-Kuwaiti Arabs	44 (30.1)	
Asians	35 (24)	
Orientals	1 (0.7)	
Caucasian	2 (1.4)	
Diagnosis		
DVT	2 (1.4)	
PE	2 (1.4)	
Unstable angina	80 (54.8)	
MI	43 (29.5)	
Stroke	4 (2.7)	
Systemic embolism	2 (1.4)	
Atrial fibrillation	9 (6.2)	
CHF	4 (2.7)	

The mean duration of UFH therapy was 3.64 days, ranging from 1 to 15 days.

### Primary Endpoints

Out of 146 patients, the number of patients who achieved the therapeutic APTT range after 6 h of UFH therapy was 39 (26.7%), whereas 60 (41.1%) patients where subtherapeutic, and 47 (32.2%) patients were over anticoagulated. At 24 h of admission, 137 patients were receiving heparin. Forty-one patients (30%) were in the therapeutic range, whereas 58 (42.3%) were subtherapeutic and 38 (27.7%) were overanticoagulated. At 48 h, 35 (33.3%) patients out of 105 who were still on heparin had a therapeutic APTT, while 49 (46.7%) patients were in the subtherapeutic range, and 20 (20%) were overanticoagulated (Table 2).

**Table 2:** APTT at 6, 24, and 48 h

APTT (Ratio)	6 h	24 h	48 h
Sub-therapeutic	60 (41.1%)	58 (42.3%)	49 (46.7%)
Therapeutic	39 (26.7%)	41 (30%)	35 (33.3%)
Over anticoagulation	47 (32.2%)	38 (27.7%)	21 (20%)
Total number	146	137	105

### Secondary Endpoints

Arabs are more likely to be subtherapeutic than non-Arabs at 6 h of starting heparin therapy (48% vs. 21%. *P*= 0.006). However, this difference disappeared at 24 and 48 h (42% vs. 41% and 48% vs. 42%, respectively).

The percentage of females who remained in the subtherapeutic APTT range after 6 h of heparin therapy was 55.3% compared with 34.7% in males (*P*= 0.003). This trend continued after 24 h (54.4% vs. 38.3%) and 48 h (53.1% vs. 44.4%).

Patients with unstable angina show more resistance to UFH than those with acute myocardial infarction in the first 48 h of UFH therapy. At 6 h of heparin therapy, 45% of patients with unstable angina were still in the subtherapeutic range compared with 20.9% of patients admitted with acute myocardial infarction (*P*= 0.004). This trend persisted at 24 and 48 h (51.3% vs. 31.6% and 53.7% vs. 34.5%, respectively).

Out of 146 patients, 88 (60.3%) had an APTT ratio below one at the time of admission, whereas 58 patients had an APTT ratio of one or more. Forty-five patients (51.1%) from the first group (with low APTT ratio) were subtherapeutic in the first 6 h after starting heparin therapy. On the other hand, out of 60 patients who were in the subtherapeutic range at 6 h of heparin therapy, 45 (75%) had an APTT ratio less than one on admission, as opposed to the 15 (25%) patients with an APTT ratio of one or more (*P*= 0.010). (Table 3)

**Table 3:** Association between baseline APTT ratio and heparin resistance at 6 h

APTT ratio on admission APTT at 6 h	APTT < 1	APTT = 1	Total
Subtherapeutic	45	15	60
Therapeutic	19	20	39
Over-anticoagulation	24	23	47
Total number	88	58	146

Bleeding attributed to UFH occurred in 8 (5.5%) patients. The bleeding episodes were epistaxis (2 cases), hemoptysis (2 cases), hematuria (1 case), localized cutaneous hematoma (1 case), bleeding from intravenous site (1 case), and gum bleeding (1 case). Most of the bleeding episodes were mild and did not lead to discontinuation of heparin, except in one patient with clinically significant hemoptysis (Table 4).

**Table 4:** Complications rate

Bleeding	8 (5.5%)
Stroke	2 (1.4%)
PE	3 (2.1%)
Extension of the thrombus	1 (0.7%)
Death	1 (0.7%)

The rate of development of recurrence of ischemia, progression of thrombus, pulmonary embolism, ischemic stroke, or death is shown in table 4.

Follow-up platelet count was not routinely ordered by treating physicians to monitor Heparin induced thrombocytopenia; it was ordered in one patient who had a complete blood count after a bleeding episode.

## Discussion

UFH is a commonly used anticoagulant in CCUs worldwide, appearing in several guidelines for the management of acute coronary syndromes and related disorders as a grade 1A recommendation[1, 7–9, 10]. The clinical effectiveness of heparin is dependent on achieving an *in vitro* defined anticoagulant effect, although there is considerable *in vivo* variation in response to a fixed dose of heparin between individuals. This is partly reflective of the pharmacokinetic limitations of heparin caused by the binding of heparin to plasma proteins, in addition to the problems encountered in measuring its desired anticoagulant effect. For these reasons, we sometimes have patients with the phenomenon known as ‘heparin resistance’. This should be taken into consideration to prevent the overadministration of heparin, with potential hemorrhagic consequences, particularly postoperatively, in cardiac bypass surgery or in acute coronary syndromes as shown in this study. In the treatment of venous thromboembolism, this phenomenon is of unclear clinical significance.

In the context of venous thromboembolism, heparin resistance is defined as the need for more than 35,000 units in a 24 h period to prolong the APTT to reach therapeutic range. In contrast, during cardiac bypass procedures the definition of heparin resistance is based on the activated clotting time (ACT), with at least one ACT less than 400 seconds after heparinization and/or the need for exogenous AT administration.

In the current study, a high rate of inadequate heparinization in patients who were in need of full anticoagulation in the initial phase of their illness was seen in 41.1% of the patients after 6 h of heparin therapy. This phenomenon had remained after 24 and 48 h of heparin therapy, putting the patients at risk of extension of their thrombotic disorder [11–14]. The potential reasons behind the high rate of inadequate heparinization in the initial phase of heparin therapy can be attributed to the unawareness of the medical personnel of heparin pharmacokinetics, leading to inappropriate initial dosing and dose adjustments. Some physicians are also overcautious while using anticoagulants because of the potential bleeding risk.

An audit of physicians' practices at three university-affiliated hospitals documented that 60% of treated patients failed to achieve an adequate APTT response (ratio of 1.5) during the initial 24 h of therapy, and that 30–40% of patients remained subtherapeutic over the next 3–4 days [3]. These problems have been alleviated with the use of protocols for the administration of heparin.

Data analysis has revealed four variables that may predict higher heparin resistance rate. The first factor is race. Arabs are more resistant to heparin therapy especially in the early phase of treatment compared with non-Arabs. This difference disappeared in subsequent days of heparin therapy. This observation needs confirmation in future studies since there has never been any similar observations in relation to heparin resistance between races in previous reports.

The second factor is gender. Females are more resistant to heparin than males in our study. The higher rate of resistance to heparin in females extended up to 48 h (Table 2). A possible explanation may be the higher level of factor VIII in females because of estrogen [8,9].

The third factor is the type of the coronary artery problem on admission. Patients who were admitted with unstable angina showed more resistance to heparin than those with acute myocardial infarction. Again, this observation has not been reported in previous reports and might be unique to the population in this study. It is possible that some physicians treated the patients with acute myocardial infarction more aggressively than they treated those with unstable angina. Another explanation is the effect of thrombolytic therapy on the coagulation system, leading to the depletion of several coagulation factors and thus resulting in less resistance to heparin therapy.

Finally, those Patients with an APTT ratio below one at presentation showed resistance to heparin therapy and were more likely to be subtherapeutic at 6 and 24 h of their admission; however, this difference disappears in the subsequent hours.

The Bleeding rate in this study was 5.5%, similar to rates in published literature. Most of bleeding episodes were minor. The rate of development of PE, progression of thrombus, ischemic stroke, and recurrence of angina were not significantly different among groups. A Lack of follow-up of platelet counts to monitor for HIT syndrome may reflect an unawareness of this phenomenon, which is associated with heparin therapy [15].

The use of evidence based Heparin protocols will help in preventing large fluctuations in APTT results and reduce the possibility of occurrence of heparin resistance in the initial phase of heparin therapy [16–19].

There is still much to learn about heparin resistance. In the context of venous thromboembolism, is heparin resistance of any clinical relevance? What is the outcome in patients treated with empirical doses of heparin when the APTT had not reached therapeutic range? In the context of cardiac bypass surgery or critical care units where activation of coagulation and fibrinolysis involve yet poorly understood mechanisms, and where postoperative bleeding complications lead to increased blood product usage and other postoperative complications, what is the optimal means of monitoring the anticoagulant and antithrombotic effect? With the recent development of new synthetic indirect and direct factor Xa inhibitors, these questions become increasingly pressing.

Low molecular weight heparin (LMWH) would be reasonable substitution for UFH because of their predicted effect and better bioavailability, as well as easy administration. LMWHs do not need laboratory monitoring, making them the drug of choice in the management of thromboembolic disorders and acute coronary syndromes [20]. Additionally, LMWHs have less plasma protein binding and are less affected by PF4.

## Conclusion

Anticoagulation with UFH in CCUs has several pitfalls that are mainly related to its properties and physicians' unawareness of its pharmacokinetics and pharmacodynamics. Adopting an approved heparin normogram and frequent monitoring of APTT, with adjustment of the dose accordingly, will result in minimization of these difficulties. Recognizing populations at risk of heparin resistance is of extreme importance and requires several studies worldwide to confirm the observed variables that may lead to heparin resistance.

## Conflict of Interest

The authors declare no conflict of interest.

## References

[1] Bertrand ME, Simoons ML, Fox KA, Wallentin LC, Hamm CW, McFadden E, De Feyter PJ, Specchia G, Ruzyllo W (2002). Management of acute coronary syndromes in patients presenting without persistent ST-segment elevation. Eur Heart J.

[2] Büller HR, Agnelli G, Hull RD, Hyers TM, Prins MH, Raskob GE (2004). Antithrombotic therapy for venous thromboembolic disease: The seventh ACCP Conference on Antithrombotic and Thrombolytic Therapy. Chest.

[3] Hirsh J, Anand SS, Halperin JL, Fuster V (2001). Guide to anticoagulant therapy: heparin: A statement for healthcare professionals from the American Heart Association. Circulation.

[4] Rauova L, Zhai L, Kowalska MA, Arepally GM, Cines DB, Poncz M (2006). Role of platelet surface PF4 antigenic complexes in heparin-induced thrombocytopenia pathogenesis: Diagnostic and therapeutic implications. Blood.

[5] Young E, Prins M, Levine MN, Hirsh J (1992). Heparin binding to plasma proteins, an important mechanism for heparin resistance. Thromb Haemost.

[6] Warkentin TE, Hayward CP, Boshkov LK, Santos AV, Sheppard JA, Bode AP, Kelton JG (1994). Sera from patients with heparin-induced thrombocytopenia generate platelet-derived microparticles with procoagulant activity: An explanation for the thrombotic complications of heparin-induced thrombocytopenia. Blood.

[7] Chew DP, Allan RM, Aroney CN, Sheerin NJ (2005). National data elements for the clinical management of acute coronary syndromes. Med J Aust.

[8] Alpert JS, Thygesen K, Antman E, Bassand JP (2000). Myocardial infarction redefined—a consensus document of the Joint European Society of Cardiology/American College of Cardiology Committee for the redefinition of myocardial infarction. J Am Coll Cardiol.

[9] Antman EM, Anbe DT, Armstrong PW, Bates ER, Green LA, Hand M, Hochman JS, Krumholz HM, Kushner FG, Lamas GA (2004). ACC/AHA guidelines for the management of patients with ST-elevation myocardial infarction: a report of the American College of Cardiology/American Heart Association Task Force on Practice Guidelines (Committee to Revise the 1999 Guidelines for the Management of Patients with Acute Myocardial Infarction). Circulation.

[10] Eikelboom J, Guyatt G, Hirsh J (2008). Guidelines for anticoagulant use in acute coronary syndromes. Lancet.

[11] Wheeler AP, Jaquiss RD, Newman JH (1988). Physician practices in the treatment of pulmonary embolism and deep-venous thrombosis. Arch Intern Med.

[12] Hull RD, Raskob GE, Brant RF, Pineo GF, Valentine KA (1997). The importance of initial heparin treatment on long-term clinical outcomes of antithrombotic therapy: The emerging theme of delayed recurrence. Arch Intern Med.

[13] Hull RD, Raskob GE, Brant RF, Pineo GF, Valentine KA (1997). Relation between the time to achieve the lower limit of the APTT therapeutic range and recurrent venous thromboembolism during heparin treatment for deep vein thrombosis. Arch Intern Med.

[14] Prandoni P, Carnovali M, Marchiori A (2004). Subcutaneous adjusted-dose unfractionated heparin vs. fixed-dose low-molecular-weight heparin in the initial treatment of venous thromboembolism. Arch Intern Med.

[15] Warkentin TE, Greinacher A (2004). Heparin-induced thrombocytopenia: Recognition, treatment, and prevention: The seventh ACCP Conference on Antithrombotic and Thrombolytic Therapy. Chest.

[16] Cruickshank MK, Levine MN, Hirsh J, Roberts R, Siguenza M (1991). A standard monogram for the management of heparin therapy. Arch Intern Med.

[17] Hull RD, Raskob GE, Rosenbloom D, Lemaire J, Pineo GF, Baylis B, Ginsberg JS, Panju AA, Brill-Edwards P, Brant R (1992). Optimal therapeutic level of heparin therapy in patients with venous thrombosis. Arch Intern Med.

[18] Robert RA, Brendan RM, Guidry JR, Fontana JR, Srinivas S (1993). The weight-based heparin dosing monogram compared with a “standard care” monogram. Ann Intern Med.

[19] Bernardi E, Piccioli A, Oliboni G, Zuin R, Girolami A, Prandoni P (2000). Monograms for the administration of unfractionated heparin in the initial treatment of acute thromboembolism: An overview. Thromb Haemost.

[20] Hirsh J, Raschke R (2004). Heparin and low-molecular-weight heparin: The Seventh ACCP Conference on Antithrombotic and Thrombolytic Therapy. Chest.

